# The effect of two QTLs for resistance to *Meloidogyne incognita* in cotton on nematode egression from roots

**DOI:** 10.21307/jofnem-2020-122

**Published:** 2021-01-13

**Authors:** Mychele B. da Silva, Richard F. Davis, Robert L. Nichols, Pawan Kumar, Peng W. Chee

**Affiliations:** 1 Formerly University of Georgia, Department of Plant Pathology, Tifton, GA 31793, currently Germains Seed Technology, Gilroy, CA 95020; 2 USDA-ARS Crop Protection and Management Research Unit, Tifton, GA 31793; 3 Cotton Incorporated, Cary, NC 27513; 4 USDA-ARS Crop Improvement and Protection Research Unit, Salinas, CA 93905; 5 University of Georgia, Department of Crop and Soil Sciences and Institute of Plant Breeding, Genetics, and Genomics, Tifton, GA 31793

**Keywords:** Cotton, *Gossypium hirsutum*, *Meloidogyne incognita*, Nematode resistance, Resistance expression

## Abstract

Cotton is widely grown in the southern US and *Meloidogyne incognita* is its most significant pathogen. The germplasm line M-120 RNR is highly resistant to *M. incognita* due to two resistance QTLs (quantitative trait loci), *qMi-C11* and *qMi-C14*. Both QTLs reduce total egg production, but the QTLs affect *M. incognita* development at different life stages. The QTLs do not appear to affect initial penetration of *M. incognita* but genotypes containing *qMi-C11* had fewer nematodes in the roots 8 days after inoculation than near isolines without *qMi-C11*, which may indicate *M. incognita* egression from roots. Three greenhouse trials were conducted using cotton isolines to determine whether *qMi-C11* and *qMi-C14* affect egression of *M. incognita* juveniles from roots. On each of the five sampling dates (4, 6, 8, 10, and 12 DAI), nematodes that egressed from roots were counted and roots were stained to count nematodes that remained in the roots. The effect of resistance QTLs on *M. incognita* egression from the roots differed among the trials. Nematode egression was consistently numerically greater, but inconsistently statistically different, from plants with both QTLs than from plants with neither QTL. Plants with only one QTL generally did not differ from plants with both QTLs, and the effects of *qMi-C11* and *qMi-C14* did not differ in any consistent way. In a separate experiment, plants with neither QTL had more eggs per egg mass than did plants with both QTLs, whereas plants with only one QTL had an intermediate number. Root gall size was measured in two trials and no consistent differences in gall size were observed. We conclude that (1) *qMi-C11* and *qMi-C14* do not stimulate nematode egression from cotton roots, (2) both *qMi-C11* and *qMi-C14* reduce *M. incognita* eggs/egg mass, and (3) neither *qMi-C11* nor *qMi-C14* affect gall size.

*Melodoigyne incognita*, the Southern root-knot nematode, causes greater total damage than any other pathogen of cotton in the United States and is responsible for losses of $147 million/year in addition to the cost of nematicides used to manage the nematode (Lawrence et al., 2015). The infective second-stage juveniles (J2) are motile and penetrate into the root system to establish feeding sites. If a feeding site is successfully established, the nematode ceases movement and progresses to subsequent developmental stages. Some plants can inhibit the establishment of *Meloidogyne* spp. feeding sites through a hypersensitive response (Davies et al., 2015), which has been suggested as a mechanism of resistance to *M. incognita* in cotton (Mota et al., 2013). Failure to establish a feeding site may lead a nematode to leave (egress from) the root (Timper et al., 2000). Nematode egression from the roots of resistant plants has been documented for peanut, potato, alfalfa, and tomato (Timper et al., 2000; Mojtahedi et al., 1988; Mullin and Brodie, 1988).

Cotton germplasm with resistance to *M. incognita* is available (Shepherd et al., 1996), and two major resistance QTLs (quantitative trait loci), *qMi-C11* and *qMi-C14*, have been identified. One QTL, *qMi-C11*, has a strong effect on root-galling and nematode reproduction, whereas the other QTL, *qMi-C14*, has little effect on the number of galls but does reduce reproduction (Gutiérrez et al., 2010; He et al., 2014). Current research utilizing isogenic lines with only one or the other resistance QTL (*qMi-C11* or *qMi-C14*) documents that *qMi-C11* inhibits successful establishment of a feeding site and subsequent development of the nematode, whereas *qMi-C14* allows the successful establishment of a feeding site but then inhibits fourth-stage juveniles (J4) from becoming adults (Da Silva et al., 2019). Inhibition of gall formation was also observed by Jenkins et al. (1995) on the M-315 RNR resistant genotype, which contains *qMi-C11* and *qMi-C14*. Successfully formed galls increased in size until 20 DAI and then ceased to enlarge further (Jenkins et al., 1995).

Since previous research found that the initial penetration of *M. incognita* into cotton roots was not affected by the *qMi-C11* and *qMi-C14* sources of resistance (Creech et al., 1995), we hypothesized that *qMi-C11*, which inhibits feeding site establishment, gall formation, and nematode development early in the infection process, leads nematodes to leave the root and may also affect gall size. The primary objective of this study was to determine whether the resistance QTLs *qMi-C11* and *qMi-C14* affect egression of *M. incognita* J2 from cotton roots. Additionally, the effect of the resistance QTLs on eggs/egg mass, percentage egg hatch, and gall size was evaluated.

## Materials and methods

The isogenic cotton lines Coker 201, M-120 RNR, CH11, and CH14 were used to evaluate the effects of *qMi-C11* and *qMi-C14* on nematode egression from roots. The germplasm line M-120 RNR was derived from backcrossing resistance to *M. incognita* (later attributed to the resistance QTLs *qMi-C11* and *qMi-C14*) into the susceptible cultivar Coker 201 (Shepherd et al., 1996). M-120 RNR contains both *qMi-C11* and *qMi-C14* (He et al., 2014). CH11 (containing *qMi-C11*) and CH14 (containing *qMi-C14*) were created by crossing M-120 RNR and Coker 201, self-pollinating plants for multiple generations, and selecting plants with either *qMi-C11* or *qMi-C14* beginning in the F2 generation. Seeds of each cotton genotype used in these studies were produced in a single batch.

Egression from roots was evaluated by planting seeds of the four isogenic lines in small tubes (Ray Leach Cone-Tainers^TM^ size RLC4; 2.5 cm × 16.1 cm) containing vermiculite and inoculating 2-week-old seedlings with 3,000 *M. incognita* J2s in a greenhouse. Each tube held one seedling. Two days after inoculation, plants were removed from tubes and roots were gently rinsed with water thereby ensuring that only nematodes inside roots remained associated with the seedlings, and then seedlings were replanted into different Cone-Tainers (size SC10; 3.8 cm  ×  21.0 cm) with fresh vermiculite thereby ensuring that any nematodes found outside the roots from that point forward must have egressed from the roots. At days 4, 6, 8, 10, and 12 after inoculation (DAI), *M. incognita* J2s were collected from vermiculite (Jenkins, 1964) and nematodes inside the roots were stained and counted using a modified version of Byrd et al. (1983). For the staining procedure, roots were carefully removed from cones, gently rinsed clean of vermiculite, soaked in a bleach solution (5.25% NaOCl) for 4 min., and then soaked in tap water for 15 min. Roots were immersed in a solution of one ml of cotton blue solution (0.35%) in 30 ml tap water and then microwaved for approximately 15 s just until the solution started to boil. Cotton blue solution was prepared by adding 0.35 g of cotton blue powder to 250 ml of lactic acid (85% (w/w) DL-lactic acid solution; 11.3 M) and 750 ml of distilled water. Roots were destained to better see nematodes inside the roots by washing stained roots in tap water and then putting them in a beaker containing glycerin. Each trial consisted of six replicates per genotype per sampling time in a randomized complete block design and the experiment was conducted three times.

Separate experiments were conducted in the greenhouse to evaluate whether the resistance QTLs influenced the number of *M. incognita* eggs per egg mass (fecundity) on the four cotton isolines. Seeds were sown in trays containing vermiculite and two-week-old seedlings were transferred (one seedling per pot) to 10.6 cm × 10.6 cm × 12.4 cm pots filled with approximately 1175 cm^3^ steam pasteurized soil (Tifton loamy sand). At the time of transfer, seedlings were inoculated with 5,000 *M. incognita* eggs produced on eggplant (the same batch of inoculum was used for all plants within a trial). The average number of eggs per egg mass was determined at 30 and 40 DAI by harvesting 10 egg masses per plant (different plants at 30 and 40 DAI), dissolving the gelatinous matrix with 0.82% NaOCl for 30 s, and counting the eggs. The four cotton genotypes were arranged in a randomized complete block design with seven replications. This experiment was conducted three times.

Percentage egg hatch (% of total eggs produced on each of the four cotton isolines) was measured to determine whether the resistance QTLs affected the viability of the eggs. Seeds of the isolines were germinated in vermiculite and two-week-old seedlings were transplanted (one seedling per pot) into 10.6 cm × 10.6 cm × 12.4 cm pots filled with steam pasteurized soil (Tifton loamy sand). Seedlings were inoculated with 7,000 *M. incognita* eggs/pot at transplanting; inoculum was produced on eggplant and a single batch of inoculum was used for an experiment. At 40 DAI, plants were removed from pots, soil was gently rinsed from roots, and roots were agitated in 0.5% NaOCl solution for 2 min to extract eggs. The eggs were then harvested on a 500 mesh sieve, rinsed with water, counted, and transferred to Kimwipes^®^ tissue placed on top of hardware cloth (0.64 cm × 0.64 cm mesh) positioned on small bowls (1.72 L) to allow egg hatch. The bowls were placed in a mist chamber for 5 days. Percentage egg hatch was calculated from the initial egg counts and the number of J2s released from the eggs after 5 days. Each trial had seven replicates per cotton genotype in a randomized complete block design, and the experiment was conducted twice.

The effects of the resistance QTLs on gall size were evaluated by comparing the size of galls produced on the four cotton isolines. Seeds of the isolines were sown (one seed per bag) in 10 cm × 15 cm  ×  0.004 cm (thickness) propylene clear bags containing vermiculite. Seven days after planting, bags were inoculated with 2,000 *M. incognita* J2, and then bags were monitored daily to detect the first appearance of root galling on any of the genotypes. Using a scanner and WinRHIZO^™^ software, gall size (area in cm^2^ as visible in the scans) was measured beginning at 10 DAI. When galling first appeared in the experiment, individual galls were labeled and those galls were measured again 7 and 14 days after the initial measurement (recorded as days 0, 7, and 14). The total number of galls measured per plant varied and ranged from one to 12. The experiment had ten replications of each cotton isoline in a randomized complete block design. Plants were grown in a growth chamber at 28^o^C with 12-hour day light per day. The experiment was conducted twice.

Data were analyzed by analysis of variance (ANOVA) using the PROC MIXED procedure in SAS 9.3 statistical software (SAS Institute Inc., Cary, NC). Cotton genotype and trial were identified as fixed effects in the analyses. No outliers were apparent, so all data were included in the analyses. Normality of the data was assumed because there were no apparent patterns to the residuals. Statistical differences among means were identified using the LSMEANS statement with the DIFF option . For the nematode egression study and the eggs per egg mass study, mean separation to identify differences among cotton genotypes were performed within a DAI. There was a significant trial × cotton genotype interaction for the nematode egression trials, so data from the trials were not combined for analysis. In contrast, there was no significant trial × cotton genotype interaction for eggs per egg mass, percentage egg hatch, or gall size, so those data were combined for analysis.

## Results

**Figure 1:**
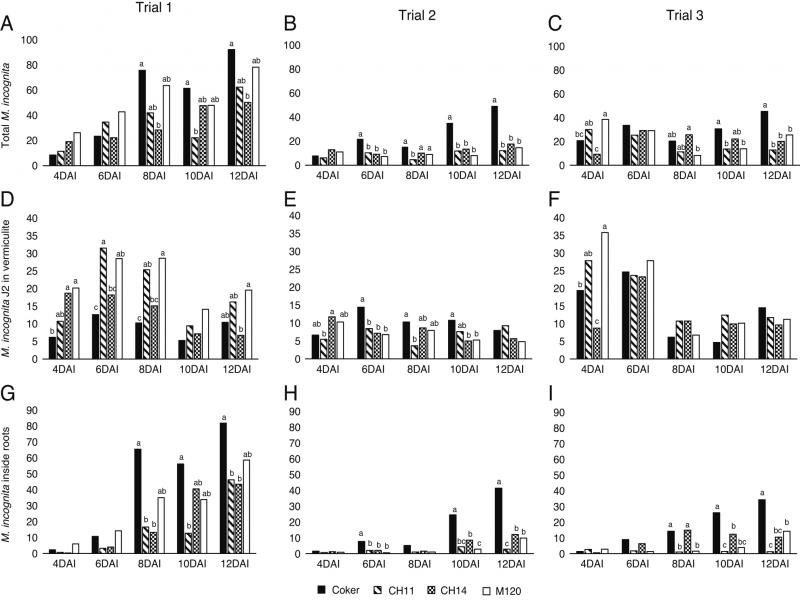
Numbers of *Meloidogyne incognita* remaining inside roots, egressed from roots into the vermiculite, and total counts on four cotton isolines that differ in *M. incognita*-resistance genes in three experimental trials. DAI = days after inoculation. Different letters over bars within a sampling date indicate significant differences at *α* = 0.05. Bars with no letters showed no significant difference within a sampling date.

In the egression study, the total number of nematodes (inside plus outside roots) did not consistently differ among the cotton isolines (Figure 1A–C), although there appeared to be a trend where Coker 201 (the susceptible standard with neither resistance QTL) consistently had numerically more nematodes than the other genotypes at 10 and 12 DAI. The total number of nematodes observed increased as Trial 1 progressed, but an increase was not observed in Trials 2 and 3. CH11 and CH14 generally did not differ from M-120 RNR in the total number of nematodes observed. Results for the number of nematodes that egressed from the roots into the vermiculite were inconsistent among trials with more nematodes egressing from the roots of M-120 RNR than Coker 201 in Trial 1 but not in Trials 2 or 3 (Figure 1D–F). CH11 and CH14 generally did not differ from M-120 RNR in the number of nematodes that egressed from the roots. There were no consistent statistical or numerical patterns among cotton genotypes for differences in the number of nematodes that egressed from the roots. The number of nematodes that remained inside the roots was greater in Coker 201 than in M-120 RNR beginning 10 DAI in Trial 2 and 8 DAI in Trial 3; although differences were not significant in Trial 1, numerical differences were consistent beginning 8 DAI (Figure 1G–I). CH11 and CH14 did not consistently differ from M-120 RNR in the number of nematodes that remained inside the roots.

**Figure 2:**
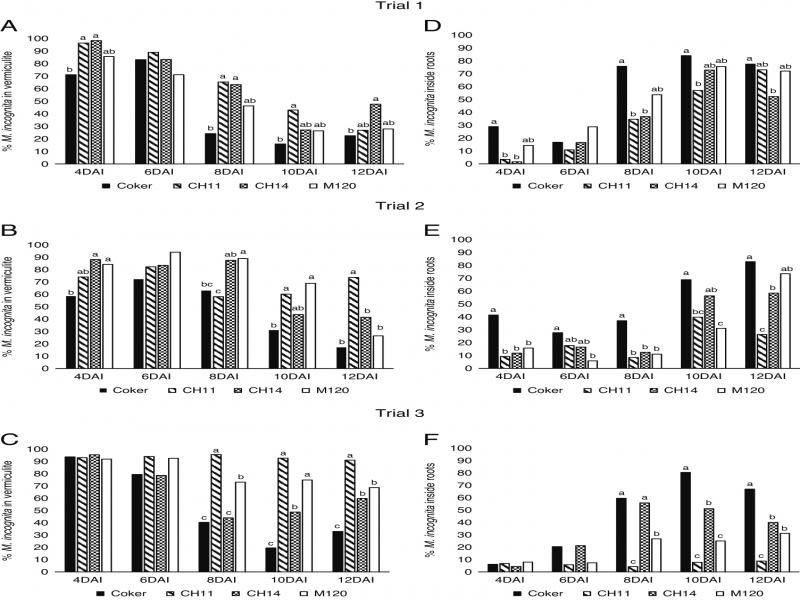
Percentage of *Meloidogyne incognita* inside and outside of the roots (egressed into the vermiculite) of four cotton isolines that differ in *M. incognita*-resistance genes in three experimental trials. DAI = days after inoculation. Different letters over bars within a sampling date indicate significant differences at *α* = 0.05. Bars with no letters showed no significant difference within a sampling date.

The number of nematodes that egressed from roots expressed as a percentage of the total number of nematodes differed among the cotton genotypes. Beginning 8 DAI, the percentage of nematodes that had egressed from the roots of M-120 RNR was generally greater than the percentage from the roots of Coker 201 with differences that were always numerically greater in all trials and generally statistically greater in Trials 2 and 3 (Figure 2A–C). The percentage of nematodes leaving the roots of CH11 and CH14 did not consistently differ from M-120 RNR among the trials. The percentage of nematodes remaining inside the roots was the inverse of the percentage egressing from roots, with Coker 201 generally retaining a greater percentage of nematodes inside the roots than did M-120 RNR and with the number of nematodes in CH11 and CH14 not consistently differing from the number in M-120 RNR (Figure 2D–F).

The number of eggs per egg mass was two to three times greater at 40 DAI than at 30 DAI on all cotton genotypes. At 30 DAI, Coker 201 had the most eggs/egg mass numerically, but only CH11 had significantly less than Coker 201 (Table 1). At 40 DAI, Coker 201 had more eggs per eggs mass than the other genotypes, M-120 RNR had the fewest, and CH11 and CH14 were intermediate. Percentage egg hatch did not differ among the genotypes and ranged from 15 to 20% (Table 1).

**Table 1.** Gall size, percentage egg hatch, and eggs/egg mass for cotton isolines that differ in *M. incognita*-resistance genes at 0, 7, and 14 days.

	Gall size (cm^2^)^1^			Hatch (%)	Eggs/egg mass	
Genotype	0 days	7 days	14 days	40 DAI^2^	30 DAI	40 DAI
Coker	0.0085 A a	0.0091 A a	0.0081 A a	17.20 A	182 A	495 A
CH14	0.0080 A a	0.0090 A a	0.0080 A a	14.96 A	123 AB	325 BC
CH11	0.0091 A ab	0.0100 A a	0.0068 A b	19.24 A	146 B	386 B
M-120	0.0056 B b	0.0074 A a	0.0075 A a	18.60 A	135 AB	300 C

1 Days after appearance of first gall. Upper case letters within a column indicate differences among genotypes, and lower case letters indicate differences among gall ages within a genotype. Different letters indicate significant differences at *α* = 0.05. ^2^DAI = days after inoculation.

The first gall size measurement (day 0) showed differences in galling among the genotypes. M-120 RNR had galls with the smallest area of 0.0056 cm^2^ and was significantly different from all the other genotypes. Gall size did not differ among genotypes on days 7 or 14. On CH11, galls were statistically larger on day 7 than on day 14 and numerically larger than on day 0. M120-RNR had smaller galls on day 0 than on days 7 and 14. The largest average gall size was on day 7 for all genotypes: gall areas on day 7 for Coker 201, CH11, CH14, and M-120 RNR were 0.009, 0.010, 0.009, and 0.007 cm^2^, respectively (Table 1).

## Discussion

One potential effect of plant resistance is the induction of nematode egression caused by the failure to establish a feeding site. Past studies observed significant egression of *M. incognita* from the roots of wild melons resistant to *M. incognita* and of *Globodera rostochiensis* from resistant potato (Faske, 2013; Mullin and Brodie, 1988). Despite some numerical trends in our studies that suggest that the susceptible Coker 201 may have had more total nematodes inside the roots than did other genotypes on later sampling dates, statistical differences were not consistent for the total number of nematodes in the roots or for the number of nematodes that egressed from the roots. These data on nematodes found in the roots are consistent with the hypothesis that initial penetration into the roots is not affected by the resistance QTLs (Creech et al., 1995). There was a consistent pattern of Coker 201 having more nematodes that remained inside the roots at later sampling dates: the pattern was numerically consistent in all three trials and was statistically significant in two of the three trials. The percentage data consistently showed that from 8 DAI onward M-120 RNR had a numerically greater percentage outside the roots than Coker 201, but the differences were not always statistically significant. Many *Globodera rostochiensis* J2 egressed from roots of both resistant and susceptible potato cultivars (Mullin and Brodie, 1988), which is similar to our observations. Our three tests of nematode egression showed large amounts of variability among tests; although the cause of the variability is not known, we speculate that the variation could have been influenced by inoculum fitness (J2 with greater or lesser energy reserves).

Although the data suggest that *M. incognita* juveniles may egress more from the roots of resistant cotton plants than from susceptible plants, the lack of consistent statistical differences forces us to conclude that nematode egression from roots does not appear to be a significant mechanism of resistance imparted by the resistance QTLs. Previous research (McClure et al., 1974) demonstrated the level of *M. incognita* egression from the moderately resistant Clevewilt-6 was similar to egression from a susceptible line. Clevewilt-6 was the source of the resistance QTL *qMi-C11* in the development of M-120 RNR (Gutierrez et al., 2010; Shepherd et al., 1996). The combination of *qMi-C11* and *qMi-C14* in M-120 RNR is epistatic for galling development (He et al., 2014). Such an epistatic effect that reduces galling has the potential to cause greater egression from M-120 RNR, however, little evidence for that was seen in our results. Despite a small difference in percentage egression from M-120 RNR compared to Coker 201 after 8 days, M-120 RNR did not differ in any consistent way from the isolines with only one QTL. Therefore, we conclude that *qMi-C11* and *qMi-C14*, either alone or in combination, have little or no effect on egression of *M. incognita* from cotton roots. A hypersensitive response is another potential mechanism of host-plant resistance (Alpizar et al., 2007; Albuquerque et al., 2010) that could be occurring in cotton with *qMi-C11* and *qMi-C14*, but we did not attempt to document hypersensitive responses in this study.

Egg masses at 40 DAI contained two to three times as many eggs as egg masses at 30 DAI. Although the difference between the susceptible and the resistant standards was not significant at 30 DAI, the susceptible cultivar had more eggs numerically and that difference increased and was significant at 40 DAI, which may suggest that the difference is a result of the rate of egg production. However, additional research is needed to determine whether differences in the number of eggs per egg mass at 40 DAI were caused by different rates of egg production, a longer period of egg production on the susceptible standard, or perhaps both. Previous research with cotton genotype carrying both *qMi-C11* and *qMi-C14* also found fewer eggs per egg mass (Creech et al., 1995), but that study was not able to test the two QTLs individually in isogenic backgrounds. Another study showed that Clevewilt-6 (the donor of *qMi-C11*) and Wild Mexican Jack Jones (the donor of *qMi-C14*) (Gutierrez et al., 2010) did not differ from a susceptible genotype in the number of eggs per egg mass after 42 DAI (Faske and Starr, 2009); although tested individually, the QTLs were not in isogenic backgrounds, which could have affected the results. Although *qMi-C11* and *qMi-C14* both reduced the number of eggs per egg mass compared to the susceptible genotype, combining *qMi-C11* and *qMi-C14* did not significantly decrease the number of eggs per egg mass compared to *qMi-C14* alone, which suggests that the effects are not additive. However, *qMi-C11* and *qMi-C14* both reduced the number of eggs per egg mass, which likely contributes greatly to the expression of resistance to *M. incognita* in cotton.

The reduction of M-120 RNR gall size may be due to the epistatic effect of *qMi-C11* and *qMi-C14*, however, galls were smaller only on day 0, which suggests that the observed difference may have been due to delayed development. A previous study with coffee plants resistant to *M. exigua* (Alpizar et al., 2007), found galls with a diameter below 1 mm (small galls) or between 1 and 3 mm (medium galls) mostly on resistant lines and galls above 3 mm only on susceptible lines. By that standard, all genotypes in our experiments presented small and medium galls with none above 3 mm regardless of resistance. M-120 RNR in our study had more small than medium galls whereas the other genotypes presented more medium sized galls. Jenkins et al. (1995) observed consistently smaller galls on M-315 RNR starting at 8 DAI than on the susceptible and partially resistant genotypes. Although our results had statistical differences only at day 0, M-120 RNR maintained small gall size (below 1 mm) throughout the experiment. Our results showed evidence that M-120 RNR reduces gall size, however it is not clear if it is due to epistatic or additive effect of the QTLs.

Percentage egg hatch did not differ among the genotypes in our study. We conclude that the QTLs that impart resistance to *M. incognita* in cotton, *qMi-C11* and *qMi-C14*, reduce the number of eggs produced but do not cause a reduction in the percentage of eggs that hatch.

Overall, this study showed that nematode egression is observed in all genotypes and does not seem to be significantly affected by resistance, that the number of eggs per egg mass contributes to the observed levels of resistance, that gall size is reduced compared to susceptible plants when both resistance QTLs are present, and that percentage egg hatch is not affected by the resistance QTLs. These finding increase our knowledge of the specific elements that contribute to *M. incognita* resistance in cotton due to the resistance QTLs *qMi-C11* and *qMi-C14.*
